# Image Non-Uniformity Correction for 3-T Gd-EOB-DTPA-Enhanced MR Imaging of the Liver

**DOI:** 10.2463/mrms.mp.2016-0012

**Published:** 2016-07-05

**Authors:** Gou Ogasawara, Yusuke Inoue, Keiji Matsunaga, Kaoru Fujii, Hirofumi Hata, Yuki Takato

**Affiliations:** 1 Department of Diagnostic Radiology, Kitasato University School of Medicine 1-15-1 Kitasato, Minami-ku, Sagamihara, Kanagawa 252-0374, Japan; 2 Department of Radiology, Kitasato University Hospital

**Keywords:** magnetic resonance imaging, non-uniformity correction, liver, 3-T, Gd-EOB-DTPA

## Abstract

**Purpose:**

Image non-uniformity may cause substantial problems in magnetic resonance (MR) imaging especially when a 3-T scanner is used. We evaluated the effect of image non-uniformity correction in gadolinium ethoxybenzyl-diethylenetriamine pentaacetic acid (Gd-EOB-DTPA)-enhanced MR imaging using a 3-T scanner.

**Methods:**

Two commercially available methods for image non-uniformity correction, surface coil intensity correction (SCIC), and phased-array uniformity enhancement (PURE), were applied to Gd-EOB-DTPA-enhanced images acquired at 3-T in 20 patients. The calibration images were used for PURE and not for SCIC. Uniformity in the liver signal was evaluated visually and using histogram analysis. The liver-to-muscle signal ratio (LMR) and liver-to-spleen signal ratio (LSR) were estimated, and the contrast enhancement ratio (CER) was calculated from the liver signal, LMR, and LSR.

**Results:**

Without non-uniformity correction, hyperintensity was consistently observed near the liver surface. Both SCIC and PURE improved uniformity in the liver signal; however, the superficial hyperintensity remained after the application of SCIC, especially in the hepatobiliary-phase images, and focal hyperintensity was shown in the lateral segment of the left hepatic lobe after the application of PURE. PURE increased LMR dramatically and LSR mildly, with no changes in CERs. SCIC depressed temporal changes in LMR and LSR and obscured contrast effects, regardless of the method used for calculation of CER.

**Conclusion:**

SCIC improves uniformity in the liver signal; however, it is not suitable for a quantitative assessment of contrast effects. PURE is indicated to be a useful method for non-uniformity correction in Gd-EOB-DTPA-enhanced MR imaging using a 3-T scanner.

## Introduction

Gadolinium ethoxybenzyl-diethylenetriamine pentaacetic acid (Gd-EOB-DTPA) is a hepatobiliary contrast agent used in magnetic resonance (MR) imaging. It is accumulated in the liver via the organic anion-transporting polypeptide of hepatocytes^1,2^ and aids the detection and characterization of focal liver lesions.^3,4^ The degree of liver parenchymal enhancement is a key parameter of diagnostic performance for focal liver lesions^5^ and correlates with liver function.^6–12^ The liver-to-muscle signal ratio (LMR), liver-to-spleen signal ratio (LSR), and contrast enhancement ratio (CER) are widely used as a quantitative indices of liver parenchymal enhancement.^6–12^

In MR imaging, spatial variations in radiofrequency transmission and reception cause non-uniformity in the signal intensity, and the image non-uniformity may pose substantial problems when a 3-T scanner is employed.^13,14^ There are various methods for non-uniformity correction.^15,16^ In this study, we evaluated two commercially available methods for non-uniformity correction, surface coil intensity correction (SCIC) and phased-array uniformity enhancement (PURE), in Gd-EOB-DTPA-enhanced imaging using a 3-T scanner. PURE is a calibration-based method that uses proton density-weighted images acquired with both the body coil and surface coil to calibrate coil sensitivity.^17,18^ SCIC is an image-based method and does not require additional data acquisition.^17^ We evaluated improvements in image uniformity and in addition, changes in quantitative indices of liver parenchymal enhancement.

## Patients and Methods

### Subjects

Twenty patients (8 men, 12 women; age 62.8 ± 15.7 years, mean ± SD) who underwent Gd-EOB-DTPA-enhanced MR imaging for clinical indications were retrospectively analyzed. The exclusion criteria were 1) poor breath holding, 2) prior liver resection, 3) prior splenectomy, and 4) severe atrophy of the erector muscles of spine, and otherwise, patients were enrolled consecutively. The criteria^2–4)^ were defined to allow appropriate setting of regions of interest (ROIs). During the enrollment, three patients were excluded due to prior liver resection. Of the 20 patients studied, 9 patients had liver cirrhosis, 3 chronic hepatitis, 2 primary biliary cirrhosis, and 6 normal background liver. The study protocol was approved by the institutional review board, and the need for informed consent was waived.

### Imaging procedures

All the examinations were performed on a 3-T clinical scanner (Discovery 750w DV25; GE Healthcare, Waukesha, WI, USA) with a 32-channel phased-array surface coil. Gd-EOB-DTPA-enhanced axial images were acquired using a T_1_-weighted three-dimensional gradient echo sequence (liver acquisition with volume acceleration [LAVA]). For dynamic imaging, 0.025 mmol/kg Gd-EOB-DTPA (Bayer Yakuhin, Osaka, Japan) was intravenously administered, and images were obtained at the precontrast, arterial, portal venous, and late phases. Hepatobiliary-phase images were acquired 20 min after contrast injection.

Typical imaging parameters for LAVA imaging were as follows: repetition time/echo time = 4.9 ms/1.8 ms, flip angle = 12°, field of view = 360 × 360 mm^2^, matrix = 320 × 192, slice thickness = 5 mm, slice number = 44, receiver bandwidth = ±83.3 kHz, and acquisition time = 16 s. True spatial resolution was 1.1 × 1.9 × 5.0 mm^3^ and reconstructed spatial resolution was 0.7 × 0.7 × 2.5 mm^3^. Field of view and slice number were increased as required in large patients. The radiofrequency transmitter was operated in the preset mode. For hepatobiliary-phase imaging, the tuning parameters (receiver gain, transmitter gain, center frequency, and gradient shim) used for dynamic imaging were manually entered to allow direct comparisons of signal intensities between dynamic and hepatobiliary-phase images. A parallel imaging technique (array spatial sensitivity encoding technique [ASSET]) was used with a reduction factor of 2.5. The calibration images were acquired with the body coil and surface coil using a three-dimensional fast spoiled gradient-recalled echo sequence, and the images acquired with the surface coil were used for ASSET reconstruction. The imaging parameters were as follows: repetition time/echo time = 1.4 ms/0.5 ms, flip angle = 1°, field of view = 480 × 480 × 480 mm^3^, matrix = 32 × 32 × 32, receiver bandwidth = ±62.5 kHz, number of excitations = 2, and acquisition time = 5s. Image non-uniformity correction was performed using the two methods provided by the manufacturer: SCIC and PURE. The uncorrected images were generated first, and non-uniformity correction was applied retrospectively. The calibration images acquired with both the body coil and surface coil were used for PURE correction.

### Visual assessment of uniformity

The uniformity of liver signal intensity was visually assessed in the precontrast and hepatobiliary-phase images. Two board-certified diagnostic radiologists independently performed visual evaluations, and the discrepancies were resolved by another board-certified diagnostic radiologist. Three image sets, uncorrected, SCIC, and PURE sets at a given phase in a given patient were simultaneously displayed on a picture archiving and communication system (PACS) viewer (EV Insite, PSP Corp., Tokyo, Japan). The observers were informed of the imaging phase and blinded to clinical information and the method for non-uniformity correction.

The observers reviewed all slices, adjusting the display scale according to their preference. They graded the superficial hyperintensity (hyperintensity near the liver surface) and focal hyperintensity as indicators of non-uniformity, using a three-point scoring system: 0 (negligible) = causing no substantial problem in assessing focal liver lesions, 1 (mild) = mildly but substantially compromising the assessment of focal liver lesions, and 2 (severe) = definitely compromising the assessment of focal liver lesions. When a focal hyperintensity score of 1 or 2 was assigned, the location of the focal hyperintensity was recorded. In addition, overall preference was determined as follows: 2 = most preferable, 1 = intermediate, and 0 = least preferable. Three different overall preference scores were assigned to the three image sets at a given phase in a given patient.

### Histogram analysis of signal intensity

We created histograms of liver signal intensities in precontrast and hepatobiliary-phase images using ImageJ software 1.49 (National Institutes of Health, Bethesda, MD, USA). Patients with focal liver lesions of 3 cm or larger were excluded from this analysis. Seven slices around the level of the porta hepatis were selected; the axial coordinate of the image was located 1 cm apart from those of the adjacent images. ROIs were manually drawn to cover the entire liver on the selected slices, and the histogram data (frequency vs signal intensity) were recorded. Manual demarcation was performed on the uncorrected images, and the same ROIs were applied to the SCIC and PURE images. The histogram data from the seven slices of a given image set were summed together, and a 15-point smoothing (simple averaging of 15 consecutive frequency values) was applied. The mode signal of the resulting histogram was determined. The signal range including the mode signal and continuously showing frequencies of more than half of the frequency at the mode were determined, and the width of this range was divided by the mode signal to determine full-width at half-maximum (FWHM). Similarly, full-width at quarter-maximum (FWQM) was determined.

### Evaluation of contrast effects

**Fig 1.**
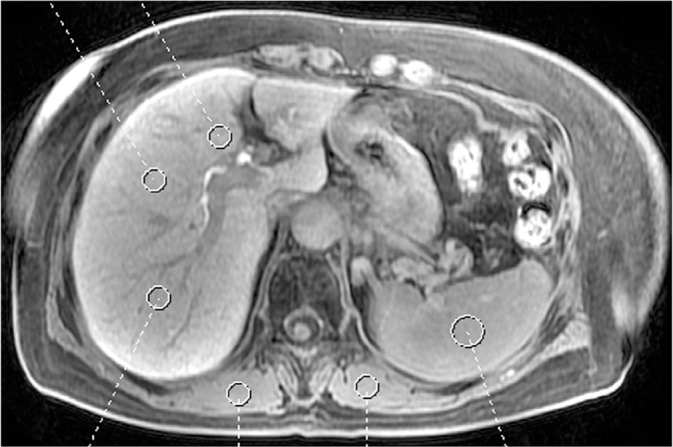
Regions of interest (ROIs) placed on the hepatobiliary-phase surface coil intensity correction (SCIC) images.

The signal intensities of the liver, muscle, and spleen were evaluated at the precontrast, arterial, portal venous, late, and hepatobiliary phases, using the PACS viewer. ROIs were first placed on the hepatobiliary-phase SCIC images. They were copied and pasted onto the dynamic SCIC images, and moved if necessary due to differences in the breath-hold positions. The locations of the ROIs were the same for each phase irrespective of the correction method. For the liver, a 100 mm^2^ circular ROI was placed in each of the anterior segment of the right hepatic lobe, the posterior segment of the right lobe, and the medial segment of the left lobe, avoiding vascular structures or tumors, on the slice that presented the right main branch of the portal vein (Fig. 1). The superficial hyperintensity on the SCIC images was also avoided to assess the signal in a major portion of the liver. Liver signal intensity was defined as the average of the mean signal intensities of the three ROIs. For muscle, 100 mm^2^ elliptical ROIs were placed in the right and left erector muscles of spine on the slice that was used to assess the liver signal. Attention was paid to minimize the inclusion of fat in the muscle ROIs. Mean signals in the right and left ROIs were averaged to determine muscle signal. For the spleen, a circular ROI of 200 mm^2^ was set at the splenic hilum level.

LMR and LSR were calculated for each image set as follows:

LMR = liver signal/muscle signal
LSR = liver signal/spleen signal

CER at each postcontrast phase was calculated from the liver signal (CER_Liver_), LMR (CER_LMR_), and LSR (CER_LSR_) as follows:

CER_Liver_ = (postcontrast liver signal − precontrast liver signal)/(precontrast liver signal)
CER_LMR_ = (postcontrast LMR − precontrast LMR)/(precontrast LMR)
CER_LSR_ = (postcontrast LSR − precontrast LSR)/(precontrast LSR).

### Statistical analysis

Visual scores were compared by the Friedman’s test, and the Wilcoxon signed rank test with Bonferroni correction was used for post hoc analysis. Cohen’s kappa statistics were calculated to assess interobserver agreement. Histogram parameters, LMR, and LSR were compared by one-way repeated analysis of variance, and post hoc analysis was performed by the paired *t*-test with Bonferroni correction. CERs were compared between uncorrected and SCIC images using the paired *t*-test. A *P*-value of <0.05 was deemed to be statistically significant.

## Results

### Visual assessment of uniformity

**Fig 2.**
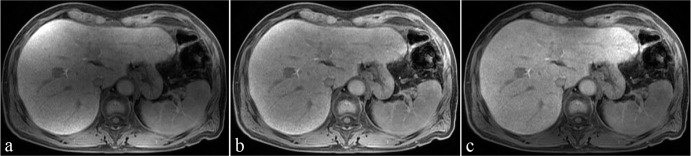
Examples of uncorrected (**a**), surface coil intensity correction (SCIC) (**b**), and phased-array uniformity enhancement (PURE) (**c**) images obtained at the hepatobiliary phase.

The superficial hyperintensity was observed in all image sets, both the precontrast and hepatobiliary-phase images of all patients, without non-uniformity correction but in no PURE image sets (Fig. 2). After the application of SCIC, the superficial hyperintensity was shown in 8 and all 20 patients for the precontrast and hepatobiliary-phase images, respectively. The visual score for the superficial hyperintensity was the highest for the uncorrected images, followed by the SCIC and PURE images, at both the precontrast and hepatobiliary phases (Table 1). The differences depending on the correction method (no correction, SCIC, and PURE) were statistically significant for all paired comparisons.

Focal hyperintensity was noted only in the PURE images (Fig. 2). It was shown in 16 and 13 patients at the precontrast and hepatobiliary phases, respectively, and was always located in the lateral segment of the left hepatic lobe. The score for focal hyperintensity was significantly higher for the PURE images than for the uncorrected and SCIC images (Table 1).

The SCIC and PURE images were judged to be the most preferable in 14 and 6 patients for the precontrast images, respectively, and in 1 and 19 patient(s) for the hepatobiliary-phase images. The uncorrected images were judged to be the least preferable in 39 of the 40 image sets including precontrast and hepatobiliary-phase image sets. The overall preference score was significantly lower for the uncorrected images than for the SCIC and PURE images at both the precontrast and hepatobiliary phases (Table 1). When the SCIC and PURE images were compared, the overall preference score was higher with SCIC at the precontrast phase and with PURE at the hepatobiliary phase; the difference was statistically significant only at the hepatobiliary phase.

Cohen’s kappa statistics indicated substantial agreement for the superficial hyperintensity (0.664) and focal hyperintensity (0.733), and almost perfect agreement for overall preference (0.875).

### Histogram analysis of signal intensity

**Fig 3.**
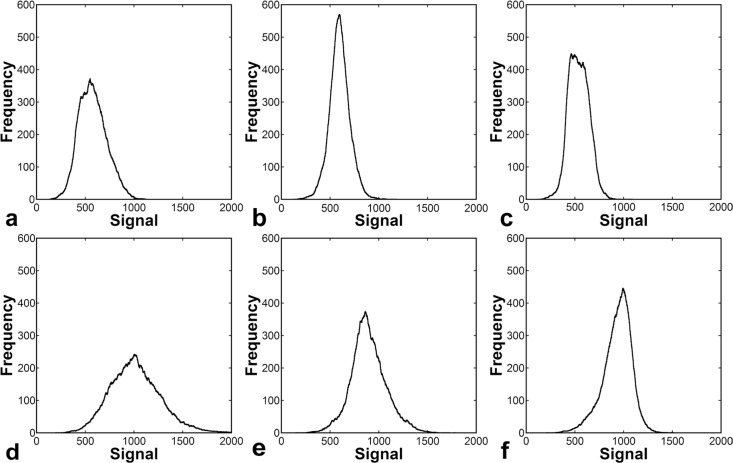
Examples of liver signal histograms created from precontrast images (**a**, uncorrected; **b**, surface coil intensity correction [SCIC]; **c**, phased-array uniformity enhancement [PURE]) and hepatobiliary-phase images (**d**, uncorrected; **e**, SCIC; **f**, PURE).

**Fig 4.**
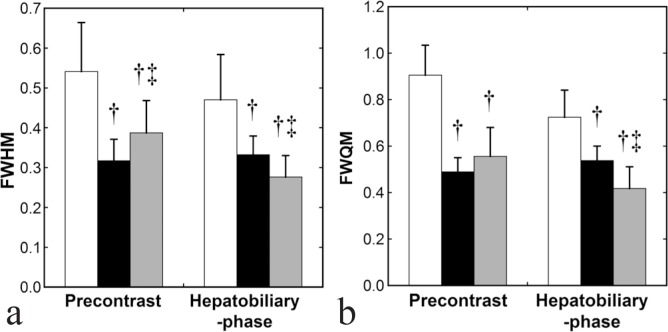
Full-width at half-maximum (FWHM) (**a**) and full-width at quarter-maximum (FWQM) (**b**) derived via histogram analysis. The white, black, and gray columns represent mean values in the uncorrected, surface coil intensity correction (SCIC), and phased-array uniformity enhancement (PURE) images, respectively. The error bars show SDs. The symbols † and ‡ indicate that a difference was significant upon comparison with uncorrected and SCIC images, respectively.

Examples of liver signal histograms are presented in Fig. 3. The frequency distribution over the signal intensity was narrower for the SCIC and PURE images than for the uncorrected images, indicating better uniformity of the liver signal. Four patients were excluded from the histogram analysis because they had large focal liver lesions. In the remaining 16 patients, FWHM and FWQM calculated from the uncorrected images were significantly larger than those calculated from the SCIC and PURE images at both the precontrast and hepatobiliary phases (Fig. 4). When SCIC and PURE were compared, the precontrast FWHM and FWQM were smaller in the SCIC images; the difference was significant for FWHM and insignificant for FWQM. In contrast, hepatobiliary-phase FWHM and FWQM were significantly smaller in the PURE images than in the SCIC images.

### Evaluation of contrast effects

**Fig 5.**
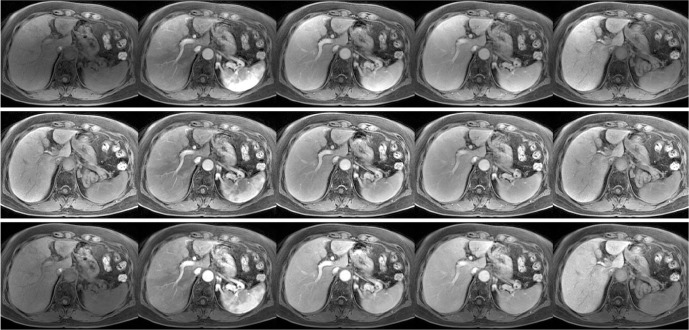
Uncorrected (upper row), surface coil intensity correction (SCIC) (middle row), and phased-array uniformity enhancement (PURE) (lower row) images of gadolinium ethoxybenzyl-diethylenetriamine pentaacetic acid (Gd-EOB-DTPA)-enhanced liver acquisition with volume acceleration (LAVA) imaging. From left to right, images at the precontrast, arterial phase, portal-venous phase, late phase, and hepatobiliary phase are presented. The display scale was adjusted for each correction method. It was not changed between phases, so that the temporal changes can be visually identified.

The PURE images demonstrated gradual increase in the liver signal and rapid increase followed by a gradual decrease in the spleen signal, as did the uncorrected images (Fig. 5). Notably, the SCIC images exhibited quite different features, and gradual liver enhancement was obscure. The liver-to-muscle and liver-to-spleen contrasts at the hepatobiliary phase were evident in the PURE images but not in the SCIC images.

**Fig 6.**
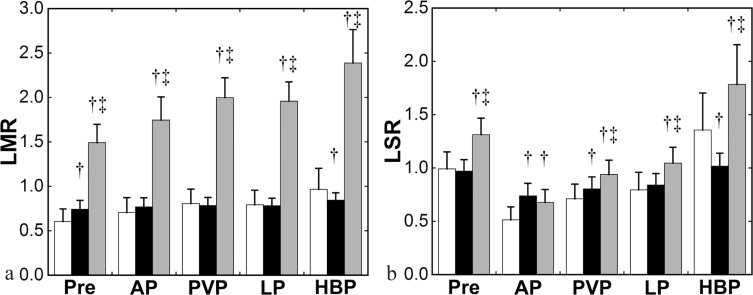
Liver-to-muscle signal ratio (LMR) (**a**) and liver-to-spleen signal ratio (LSR) (**b**). The white, black, and gray columns represent mean values in the uncorrected, surface coil intensity correction (SCIC), and phased-array uniformity enhancement (PURE) images, respectively. Pre, AP, PVP, LP, and HBP indicate precontrast, arterial, portal venous, late, and hepatobiliary phases, respectively. The error bars show SDs. The symbols † and ‡ indicate that a difference was significant upon comparison with uncorrected and SCIC images, respectively.

Non-uniformity correction greatly influenced the estimates of LMR and LSR (Fig. 6). For the LMR, gradual increase was observed in the uncorrected and PURE images, and the PURE images yielded a much larger values at each phase. LMR in the SCIC images remained relatively constant throughout the time course, and was significantly larger at the precontrast phase and significantly smaller at the hepatobiliary phase, compared with that in the uncorrected images. LSR in the uncorrected images decreased early after contrast injection, reflecting stronger enhancement in the spleen than in the liver, and increased at the hepatobiliary phase. PURE yielded significantly larger values at all the phases. Temporal changes in LSR were less apparent with SCIC. LSR in the SCIC images was significantly larger at the arterial and portal-venous phases and significantly smaller at the hepatobiliary phase, compared with that in the uncorrected images.

**Fig 7.**
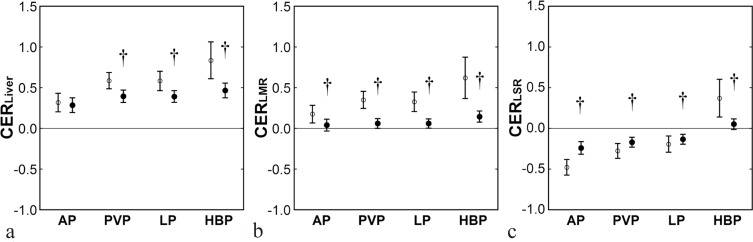
CER_Liver_ (**a**), CER_LMR_ (**b**), and CER_LSR_ (**c**). The open and closed circles represent mean values in the uncorrected and SCIC images, respectively. AP, PVP, LP, and HBP indicate arterial, portal venous, late, and hepatobiliary phases, respectively. The error bars show SDs. The symbol † indicates that a difference was significant upon comparison with uncorrected images.

CERs were theoretically identical between the uncorrected and PURE images because the same correction maps were applied to all phases to create PURE images from the uncorrected images. After confirming this using actual data, we performed further analyses regarding CERs calculated from the uncorrected and SCIC images. Without non-uniformity correction, CER_Liver_ clearly demonstrated increasing enhancement of the liver parenchyma (Fig. 7). CER_LMR_ and CER_LSR_ were smaller than CER_Liver_ due to enhancement in the muscle and spleen. CER_LSR_ was negative in the dynamic images, reflecting stronger enhancement in the spleen than in the liver. The SCIC images yielded notably different results. CER_Liver_ was definitely smaller in the SCIC images than in the uncorrected images, with statistical significances, except at the arterial phase. At all phases, CER_LMR_ was close to zero and was significantly smaller than in the uncorrected images. CER_LSR_ tended to converge to zero and were significantly larger in the dynamic images and significantly smaller in the hepatobiliary-phase images, compared with the uncorrected images.

## Discussion

In this study, we evaluated image non-uniformity in Gd-EOB-DTPA-enhanced MR images obtained using a 3-T scanner and demonstrated hyperintensity near the liver surface in the absence of non-uniformity correction. Histogram analysis, in addition to visual assessment, was performed to objectively evaluate non-uniformity in the liver signal. To evaluate the liver comprehensively, ROIs were drawn over the entire liver on the slices selected for analysis. Although the patients with massive liver tumors were excluded from the histogram analysis, it was inevitable to include intrahepatic vessels in the ROIs. The low-signal tail of the histogram should have been affected by pixels representing intravascular signals entirely or partially. To minimize the influence of intrahepatic vessels, the FWHM and FWQM of the histogram were determined from the data around the mode signal and were used as indicators of uniformity.

PURE is a calibration-based method for non-uniformity correction and employs calibration images, similarly with a Prescan Normalize (Siemens) and CLEAR (Philips).^17,18^ Compared to no correction, the application of PURE increased the overall preference scores on visual assessment and decreased FWHM and FWQM on histogram analysis, indicating an improvement of uniformity in the liver signal. The superficial hyperintensity disappeared; however, focal hyperintensity often appeared in the lateral segment of the left hepatic lobe. When the display scale is optimized to visualize a large portion of the liver, including the right lobe and left medial segment, a part of the left lateral segment may become too bright on the display. PURE is indicated to attain favorable non-uniformity correction; however, additional assessment after readjustment of the display scale may be required for the evaluation of the left lateral segment.

A calibration-based method requires a calibration scan and may be affected by the spatial mismatch between the images to be corrected and the calibration images. SCIC, an image-based method, does not use calibration images,^17^ and errors due to spatial mismatch do not occur. In this study, SCIC improved uniformity in the liver signal, yielding higher overall preference scores and smaller FWHM and FWQM compared with no correction. However, the superficial hyperintensity was shown on visual assessment. It was more evident in the hepatobiliary-phase images than in the precontrast images. The evaluation of the overall preference score and histogram parameters also suggested that SCIC is more suited for the correction of the precontrast images than that of the hepatobiliary-phase images. SCIC may be prone to cause artificial hyperintensity near the liver surface when the contrast between the liver and surrounding areas is high.

Various indices have been used for a quantitative assessment of liver parenchymal enhancement in Gd-EOB-DTPA-enhanced MR imaging. LMR and LSR represent the liver signal normalized for the muscle signal and spleen signal, respectively.^6–8^ CER can be calculated from the liver signals with^8–10^ or without^8,10–12^ such normalization. We set the liver ROIs avoiding the superficial hyperintensity in the SCIC images; as a result, the liver ROIs tended to be located deeper than the muscle and spleen ROIs. LMR was much larger with PURE than without non-uniformity correction probably because the superficial location of the muscle ROI caused overestimation of the muscle signal and, consequently, underestimation of LMR in the uncorrected images. PURE increased LSR mildly, which appears to be attributable to the more superficial location of the spleen ROI compared with the liver ROI. PURE appears to aid the assessment of image contrasts between organs.

In contrast to PURE, SCIC failed to recover LMR and LSR. Although PURE applies the same correction maps to different image sets within an examination and thus does not affect CERs, SCIC determines actual correction for each image in serial imaging and may not preserve temporal changes in the signal induced by contrast injection. Temporal changes in LMR and LSR, shown with no correction, was definitely reduced, and neither liver-to-muscle nor liver-to-spleen contrast was evident at the hepatobiliary phase. The gradual increase in CER calculated directly from the liver signal was obscured, and those calculated from the LMR and LSR remained approximately to be zero, apparently suggesting a lack of substantial contrast enhancement. SCIC decreases non-uniformity in signal intensity without measuring non-uniformity in the system sensitivity. SCIC may mistake true signal differences as false differences, thus reducing true image contrasts between organs and between phases. Care should be taken when evaluating abdominal MR images corrected with SCIC. This method cannot be used for a quantitative evaluation of Gd-EOB-DTPA-enhanced MR images.

The main purpose of Gd-EOB-DTPA-enhanced MR imaging is to evaluate focal liver lesions. The usefulness of non-uniformity correction for that purpose remains to be investigated. The image non-uniformity may affect the evaluation of focal liver lesions through its effect on determination of display conditions. A wide display window may be preferred to simultaneously present the whole liver in non-uniform images, which reduces visual lesion contrast. Instead, a radiologist may use a narrow window and change the window level in observing hyperintense regions, which may increase the time required for image reading. When investigating the usefulness of non-uniformity correction in lesion evaluation, the strategy of determining the display conditions should be predefined.

## Conclusion

In Gd-EOB-DTPA-enhanced MR imaging using a 3-T scanner, image non-uniformity influences visual and quantitative evaluation. SCIC, an image-based correction technique, improves uniformity but causes superficial hyperintensity, especially in the hepatobiliary-phase images. SCIC does not recover contrast among organs and obscures contrast effects in liver parenchyma. PURE, a calibration-based technique, achieves relatively favorable non-uniformity correction, although artificial focal hyperintensity may be observed in the lateral segment of the left hepatic lobe.

## Figures and Tables

**Table 1.** Visual scores

	Uncorrected	SCIC	PURE
Superficial hyperintensity			

Precontrast	1.90 ± 0.31	0.40 ± 0.50†	0.00 ± 0.00†‡
Hepatobiliary	1.95 ± 0.22	1.20 ± 0.41†	0.00 ± 0.00†‡

Focal hyperintensity			

Precontrast	0.00 ± 0.00	0.00 ± 0.00	1.15 ± 0.75†‡
Hepatobiliary	0.00 ± 0.00	0.00 ± 0.00	0.85 ± 0.75†‡

Overall preference			

Precontrast	0.00 ± 0.00	1.70 ± 0.47†	1.30 ± 0.47†
Hepatobiliary	0.05 ± 0.22	1.00 ± 0.32†	1.95 ± 0.22†‡

Data are mean ± SD.

The symbols † and ‡ indicate that a difference was significant upon comparison with uncorrected and surface coil intensity correction (SCIC) images, respectively. PURE, phased-array uniformity enhancement
